# Theory, modelling and observations of marginal ice zone dynamics: multidisciplinary perspectives and outlooks

**DOI:** 10.1098/rsta.2021.0265

**Published:** 2022-10-31

**Authors:** Luke G. Bennetts, Cecilia M. Bitz, Daniel L. Feltham, Alison L. Kohout, Michael H. Meylan

**Affiliations:** ^1^ School of Mathematical Sciences, University of Adelaide, Adelaide, South Australia 5005, Australia; ^2^ Department of Atmospheric Sciences, University of Washington, Seattle, WA, USA; ^3^ Department of Meteorology, University of Reading, Reading, UK; ^4^ National Institute of Water and Atmospheric Research, Christchurch, New Zealand; ^5^ School of Mathematical and Physical Sciences, University of Newcastle, Callaghan, New South Wales 2308, Australia

**Keywords:** marginal ice zone, dynamics, sea ice, ocean waves, wave–ice interactions, ice floes

## Abstract

The marginal ice zone (MIZ) is the dynamic interface between the open ocean and sea ice-covered ocean. It is characterized by interactions between surface gravity waves and granular ice covers consisting of relatively small, thin chunks of sea ice known as floes. This structure gives the MIZ markedly different properties to the thicker, quasi-continuous ice cover of the inner pack that waves do not reach, strongly influencing various atmosphere–ocean fluxes, especially the heat flux. The MIZ is a significant component of contemporary sea ice covers in both the Antarctic, where the ice cover is surrounded by the Southern Ocean and its fierce storms, and the Arctic, where the MIZ now occupies vast expanses in areas that were perennial only a decade or two ago. The trend towards the MIZ is set to accelerate, as it reinforces positive feedbacks weakening the ice cover. Therefore, understanding the complex, multiple-scale dynamics of the MIZ is essential to understanding how sea ice is evolving and to predicting its future.

This article is part of the theme issue ‘Theory, modelling and observations of marginal ice zone dynamics: multidisciplinary perspectives and outlooks’.

The profound physical transformations to Arctic [1] and Antarctic [2] sea ice covers caused by climate change have driven an explosion in research activity on the marginal ice zone (MIZ), particularly the dynamic (and thermodynamic) feedback processes occurring in the region. Fundamental research questions are being addressed, such as the rate at which the MIZ ice cover attenuates ocean waves, the threshold for waves to break up consolidated ice covers, the typical floe size distributions encountered in the MIZ and the dynamic response of the granular ice cover to forcing from winds and currents. The field of MIZ dynamics is moving rapidly due to advances across a range of physical, mathematical, earth and engineering sciences, often revolving around large multi-national research programmes and experimental campaigns. Progress is beginning to translate into predictions of the MIZ’s role in the response of sea ice to warming temperatures and its impact on other components of the atmosphere–ice–ocean–land feedback system.

After approximately a decade of sustained advances in MIZ dynamics, the current theme issue brings together many of the most prominent research communities involved to showcase the state of the art in theory, modelling and observations. Beyond presenting the latest research advances, the theme issue places emphasis on communicating important new findings to the broader research community and highlighting the critical role of the MIZ in the Earth system. Moreover, the theme issue aims to delineate pathways for the field to create more significant impact and improve understanding of climate change, noting that MIZ researchers are demonstrating strong commitment to integrate MIZ-specific physics into relevant components of Earth-system models used by influential scientific bodies, such as the International Panel on Climate Change.

The theme issue is dedicated to the career of Prof. Vernon Squire following his retirement in 2020. Squire was a leading member of the University of Cambridge’s Scott Polar Research Institute (SPRI) team that conducted pioneering Arctic MIZ experiments in the 1970s and 1980s [3] and participated in large international experiments [4,5], during an era in which the Arctic Ocean was a potential battleground for the reigning superpowers. As part of the SPRI team, Squire helped develop seminal theories for wave attenuation in the MIZ [6] and wave-induced ice breakup [7], alongside cognate work on iceberg dynamics [8], loads imposed on floating ice by vehicles [9] and more. He moved to the University of Otago, New Zealand, in 1987, where he stayed for the remainder of his career, leading a small but productive research team in the Department of Mathematics and Statistics, which was largely responsible for carrying the field of MIZ dynamics forward until the early 2010s, and (directly or indirectly) spawned many members of the present research community. His team is best known for developing the theories of wave–ice interactions that underpin the present state of the art, but were also active in remote sensing [10] and laboratory experimental modelling [11,12], both of which have become active research areas. Squire wrote two highly cited review articles during this period, *Of Ocean Waves and Sea Ice* [13] and *Of Ocean Waves and Sea Ice Revisited* [14], which communicated the field to a broad audience in his hallmark engaging and (to appropriate one of his favourite words) mellifluous writing style. He came into the recent research boom recognized as one of the most prominent figures in MIZ dynamics and has been influential in the rapid development of the field through his involvement in international programmes, such as the Norwegian-led *Waves in Ice Forecasting for Arctic Operators* [15] and the US-led *Sea State and Boundary Layer Physics of the Emerging Arctic Ocean* [16], co-organizing the *Mathematics of Sea Ice Phenomena* research programme at the Isaac Newton Institute [17], and promoting the progress of the field through perspectives and review articles [18], including a third instalment in the *Of Ocean Waves and Sea Ice* series [19]. The guest editors and authors express their appreciation for his enthusiasm and mentorship, and are delighted to offer the theme issue to celebrate his contributions. Prof. Squire is actively involved in the theme issue as the author of a preface [20] and a synopsis of the research presented in the theme issue that includes directions for future interdisciplinary research [21].

The main body of the theme issue is divided into three focus topics that encapsulate current trends in multidisciplinary MIZ-dynamics research. Each topic opens with a mini-review that introduces the topic and highlights implications for other areas of science, followed by three to four research articles that collectively cover the latest activity in theory, modelling and observations within the topic area. Focus topic I is on wave propagation in the MIZ: the mini-review by Thomson [22] highlights debates in the community on whether wave scattering or viscous dissipation dominates wave attenuation in the MIZ and the potential coastal impacts of waves with a weakened sea ice buffer; Shen [23] compares the leading theories for wave propagation in the MIZ; Toffoli *et al.* [24] analyse experimental laboratory models of irregular wave interactions with artificial floes, focusing on nonlinear interaction processes; Waseda *et al.* [25] present multiple-platform field observations of waves and ice in the MIZ; and Perrie *et al.* [26] implement a new source term for wave–ice interactions in a numerical wave forecasting model. Focus topic II is on floe size distributions: the mini-review by Horvat [27] summarizes the history of floe size distribution (FSD) observations and models, and discusses the climate-scale impacts of the MIZ FSD; Montiel & Mokus [28] provide new theoretical support for lognormal FSDs in the MIZ; Hwang & Wang [29] use satellite observations in a region of the Arctic MIZ to study the FSD life-cycle; and Cooper *et al.* [30] compare *in situ* wave observations with outputs from coupled wave and sea ice models, in which the sea ice model contains a recently developed FSD sub-model. Focus topic III is on ice dynamics and breakup: the mini-review by Dumont [31] gives an overview of the current state of the art in the area; Herman [32] uses discrete-element simulations to conduct a theoretical analysis of the MIZ rheology; Boutin *et al.* [33] compare MIZ extents from a coupled wave–ice numerical model involving a rheology sub-model to satellite observations; and Auclair *et al.* [34] use a numerical model to investigate the effects of wind and wave radiative stresses on ice dynamics. The theme issue closes with two future-facing articles: the first is the above-mentioned synopsis by Squire [21]; and the second article is by the guest editors [35] and generates visions for the future of the field and pathways to create impact in the broader science community, e.g. impacts on climate science.

## Data Availability

This article has no additional data.
